# Pattern of Bacterial Flora and Effect of Disinfection on Fingerprinting Biometric Devices at a Tertiary Care Health Facility: An Interventional Study

**DOI:** 10.7759/cureus.64718

**Published:** 2024-07-17

**Authors:** Mudit Chauhan, Naresh P Singh, Amit Singh, Ankita Sharma, Pankaj Jain, Pooja Pathak

**Affiliations:** 1 Community Medicine, Baba Raghav Das Medical College, Gorakhpur, IND; 2 Community Medicine, Uttar Pradesh University of Medical Sciences, Etawah, IND; 3 Microbiology, Uttar Pradesh University of Medical Sciences, Etawah, IND; 4 Community Medicine, Maharshi Vishwamitra Autonomous State Medical College, Ghazipur, IND

**Keywords:** health care provider, tertiary health care, disinfection, bacterial flora growth, biometric fingerprint devices

## Abstract

Introduction

Recently, many public and private sector institutions and hospitals have installed biometric fingerprint devices for attendance purposes. This step is taking us toward modernization but biometric devices have their cons and pros; if not sterilized at regular intervals, then it may be a potent cause of transmission of various infections. Many studies have reported the presence of coagulase-negative *Staphylococcus aureus* (CONS), methicillin-resistant *Staphylococcus aureus* (MRSA), *Escherichia coli*, *Pseudomonas*, and others.

Aim

To study the pattern of bacterial flora and the effect of disinfection on fingerprinting biometric devices at a tertiary care health facility.

Materials and methods

A total of 138 biometric devices were used, out of which 105 were frequently (at least 50 uses per day) used and functional. So, 105 samples were collected on day zero (baseline), of which 43 and 62 were from clinical and non-clinical groups, respectively. The devices were disinfected with isopropyl alcohol (w/v 70%) and subsequent samples were taken on day 1 (after 24 hours) and day 7. The samples were collected and transported to the microbiology lab for culture and incubation.

Statistical analysis was performed using SPSS software version 25 (IBM Corp., Armonk, NY) employing chi-square, Cochran’s Q test, and post hoc test. A p-value ≤0.05 at a 95% confidence interval was considered to be statistically significant.

Results

At baseline (day 0), bacterial growth was observed in 13 (38%) devices from the clinical group and 10 (20%) from the non-clinical group. After disinfection with 70% isopropyl alcohol, bacterial growth was reduced by 83% on day 1 but increased by 82% on day 7. These changes were statistically significant (p ≤ 0.05).

Conclusion

The present study concluded the definite presence of bacterial flora on the biometric fingerprint devices which are prone to carry and transmit microorganisms indirectly from person to person. The surface of biometric fingerprinting devices should be disinfected daily. If not possible, it should be done on an average of every third day to control and minimize the transmission of microorganisms.

## Introduction

Nowadays, most of the health institutions and hospitals in public and private sectors have installed biometric fingerprint devices (BFDs) on their premises to record the attendance of their staff [1]. This is a step towards modernization, but it is associated with some disadvantages too. These biometric devices, if not sterilized at regular intervals, may become a potent site and cause of transmission of various infections among staff members, their families, and indirectly to the community and subsequently bringing back the infection to the hospital premises. This vicious cycle will keep on occurring if proper measures are not taken at frequent intervals.

As a part of administrative measures to ensure the presence of employees at their respective workstations, everybody is imposed to use the BFD, at least once or twice a day, to record attendance which can be a potential source of transmission. The surface of biometric devices installed at various health-related as well as non-health-related facilities may harbour a variety of potentially pathogenic bacteria namely coagulase-negative *Staphylococcus aureus* (CONS), methicillin-resistant *Staphylococcus aureus* (MRSA), Gram-negative bacilli, *Enterobacter* species, *Escherichia coli*, *Pseudomonas*, and many more. Many studies have even reported the presence of highly drug-resistant bacteria qualifying to superbug and ESKAPE group (*Enterococcus faecium*, *Staphylococcus aureus*, *Klebsiella pneumoniae*, *Acinetobacter baumannii*, *Pseudomonas aeruginosa,* and *Enterobacter* spp) [2].

Most of these organisms can survive in a dry surface environment which eventually may become the source of transmission. Most of these organisms survive on the hands for more than 30 minutes, which is the crucial time to break the chain of transmission which can be done by performing adequate and proper hand hygiene. Bacterial pathogens especially on non-living objects can survive for days to weeks, acting as exogenous sources of infection in hospital-acquired infection [3,4].

Given these concerns, this study aims to systematically investigate the presence and pattern of bacterial flora on BFDs used in a tertiary healthcare setting. Furthermore, it seeks to evaluate the effectiveness of 70% isopropyl alcohol as a disinfectant in reducing bacterial contamination on these devices. By assessing bacterial growth before and after disinfection, the study intends to determine the optimal frequency of disinfection required to minimize the risk of infection transmission among healthcare workers and support staff [5,6].

## Materials and methods

This hospital-based interventional study was conducted at a tertiary-level teaching institution with an associated hospital providing health care in a rural setting, from August 2019 to November 2019. This institution had an overall strength of 5000 employees and students; approximately 1500 were regular salaried employees and nearly 3500 students pursuing various academic courses in medicine, nursing, pharmacy, para-medical sciences, and dentistry. To record their daily attendance, a total of 138 biometric devices have been installed in the institution at various sites, out of which, 105 devices were sampled for study intervention as the remaining devices were either non-functional or rarely used because of their inconvenient location of installation. The research team comprised faculty members, postgraduate students from the Department of Community Medicine and Microbiology, and lab technicians to help in the intervention procedure and data collection.

Following were the steps of data collection and intervention:

- Swab samples were collected from the surface of all the selected biometric devices (in total 105 devices were functional and recording the attendance of at least 50 or more users) on the first day of intervention (day zero/baseline), of which, 47 samples were from the hospital block (clinical setting group). The remaining 58 samples were collected from the academic and administrative block (non-clinical setting group).

- After obtaining these baseline samples, all biometric devices’ surfaces were disinfected with isopropyl alcohol (70% w/v concentration). Repeat swab samples were collected from all the biometric devices after 24 hours, that is, on day 1 and again on day 7.

- Swab samples were collected using sterile cotton swabs soaked in 2 ml of distilled water, swabbing a 4 cm² area for 10 seconds, and immediately transported to the microbiology lab of the tertiary care health facility at the study place.

- The culture was done on blood agar and MacConkey agar plate by the experts in the microbiology department and subsequently incubated overnight at 37˚C for the isolation of bacterial growth.

-Similarly, the repeat samples taken on day 1 and day 7 were subjected to culture and sensitivity to determine the efficacy of disinfectant about changes in the bacterial load and time interval required for the desired effect. The biometric devices that were either non-functional or rarely used were excluded from the sample collection.

Statistical analysis

The data collected was scrutinized for completeness and entered in a Microsoft Excel spreadsheet and statistically analysed using SPSS software version 25 (IBM Corp., Armonk, NY) using the chi-square test, Cochran’s Q test, and post hoc test. At a 95% confidence interval, a p-value ≤0.05 was considered to be statistically significant.

## Results

The present study was conducted on a total of 105 functional biometric sites which were categorized into clinical group (n=47) and non-clinical group (n=58) based on their installed location in the healthcare facility where this study was performed. Culture reports of the swab samples collected from these sites revealed an overall proportion of bacterial growth on 23 devices (22%). It was also observed that 13 (12.4%) clinical group BFDs and 10 (9.6%) non-clinical group devices yielded growth at baseline (day zero) of the study period (Table 1). This observed difference in the growth of bacterial flora on the surface of biometric devices at two different study sites was not found to be statistically significant (p=0.841 at 95% CI). It was observed that among the positive bacterial growths reported on the culture plates, CONS was 8 (7.6%), MRSA was 2 (1.9%), *Pseudomonas aeruginosa* 3 (2.9%), *Escherichia coli* 4 (3.9%), *Enterococcus faecalis* 3 (2.9%), *Enterobacter* spp. 2 (1.9%), and *Acinetobacter* spp. 1 (0.9%) on baseline (day zero) of intervention as depicted in Table 2. After the intervention, that is, disinfecting the surface of BFDs with isopropyl alcohol (w/v 70%), the overall bacterial flora growth on the devices was remarkably reduced from 23 to four, i.e., 82.6% on day 1, while the proportional reduction in bacterial growth on day 7 after the intervention was 17.4% only (from 23 to 19). CONS and *Escherichia coli* were eliminated in 75% (reduced from 8 to 2) of the positive samples, *Enterococcus faecalis* in 66.7% (reduced from 3 to 1) of samples while MRSA, *P. aeruginosa*, *Enterobacter* spp., and *Acinetobacter* spp. showed a 100.0% reduction (reduced from 2 to 0, 3 to 0, 2 to 0, and 1 to 0, respectively) after disinfecting with isopropyl alcohol (w/v 70%). All the identified bacteria except *Acinetobacter* spp. showed a resurgence in samples collected for culture after seven days of intervention (Table 2).

**Table 1:** Presence of bacterial flora on the biometric fingerprint devices (N=105) at baseline day 0, day 1, and day 7 N = total no. of biometric devices; n = subparts of N; X^2 ^=* *chi-square test value; p-value ≤0.05 = significant; p-value ≥0.05 = insignificant.

S. No.	Biometric sites		Status of bacterial flora		Statistical interpretation
			Growth present n (%)	Growth absent n (%)	
1.	(Baseline Day 0)	Clinical (n=47)	13 (27.7)	34 (72.3)	χ^2^= 1.647, p-value = 0.199
		Non-clinical (n=58)	10 (17.2)	48 (82.8)	
		Total (N=105)	23 (22)	82 (78)	
2	Day 1	Clinical (n=47)	3 (6.3)	44 (93.6)	χ^2^= 1.437, p-value = 0.231
		Non-clinical (n=58)	1 (1.8)	57 (98.2)	
		Total (N=105)	4 (3.8)	101 (96.3)	
3	Day 7	Clinical (n=47)	13 (27.7)	34 (72.3)	χ^2^= 6.151, p-value = 0.013
		Non-clinical (n=58)	6 (10.3)	52 (89.7)	
		Total (N=105)	19 (18.1)	86 (89.9)	

The proportion of growth of micro-organisms reduced from 23 (22%) to 4 (3.8%) on day 1, and a further increase was observed on day 7 samples 19 (18.1%). Statistical comparison of growth results on three occasions, namely day zero, day 1, and day 7 of interventions using Cochran’s Q test revealed that the difference between the three occasions was statistically significant (p=0.001).

**Table 2:** Pattern and effect of the intervention on bacterial flora on biometric devices (N=105) N = total no. of biometric devices; n = subparts of N; X^2 ^= chi-square test value; p-value ≤0.05 = significant; p-value ≥0.05 = insignificant.

Name of the pathogen detected	Intervention			Statistical interpretation
	Baseline Day 0 n (%)	Day 1 n (%)	Day 7 n (%)	
No pathogen detected	82 (78.0)	101 (96.3)	86 (81.9)	-
Coagulase-negative *Staphylococcus* spp. (CONS)	8 (7.6)	2 (1.9)	7 (6.7)	-
Methicillin-resistant *Staphylococcus aureus* (MRSA)	2 (1.9)	0 (0.0)	1 (0.9)	-
Pseudomonas aeruginosa	3 (2.9)	0 (0.0)	4 (3.9)	-
Escherichia coli	4 (3.9)	1 (0.9)	5 (4.8)	-
Enterococcus faecalis	3 (2.9)	1 (0.9)	1 (0.9)	-
*Enterobacter* spp.	2 (1.9)	0 (0.0)	1 (0.9)	-
*Acinetobacter* spp.	1 (0.9)	0 (0.0)	0 (0.0)	-
Proportion of growth on the days of observation	23 (22.0)	4 (3.8)	19 (18.1)	-
Proportional reduction in bacterial flora after intervention with reference to baseline status (n=23)	--	19 (82.6)	4 (17.4)	-
Cochran’s Q test				Cochran's Q=14.195, p= 0.001
Post hoc test (between groups)	Baseline day 0 vs day 1			X^2^=15.34, p<0.001
	Day 0 vs day 7			X^2^=0.758, p=0.384
	Day 1 and day 7			X^2^=9.95, p=0.002

**Figure 1:**
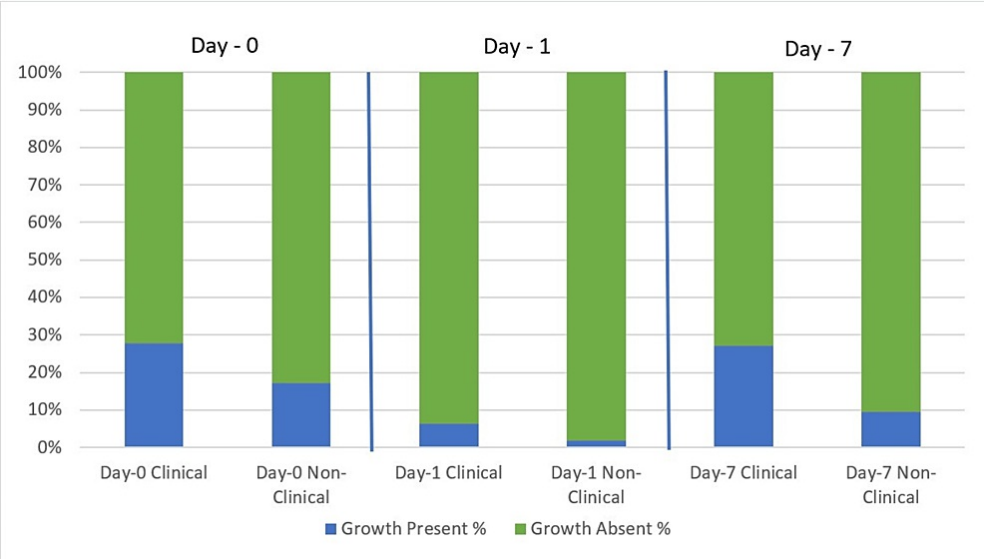
Growth of bacterial flora on clinical and non-clinical biometric fingerprinting devices

Post hoc test was done to evaluate the inter-group variability and it revealed that the proportional difference was statistically significant for baseline day zero versus day 1 results (p<0.001) and day 1 versus day 7 finding (p=0.002), while it was not significant for baseline day zero versus day 7 (p=0.384) (Figure 1).

## Discussion

The present interventional study revealed that the overall bacterial culture positivity rate of all the samples taken from clinical and non-clinical groups of BFDs was 23 (22.0%) with a marginally higher proportion in clinical settings as compared to non-clinical settings. A similar study conducted by S. Nirupa et al. at a tertiary care health facility in southern India reported a positivity rate of 46% (39), while another study conducted by S. Nancy et al., in central India, found that the surface of 10 (33.3%) biometric devices was culture positive [7,8]. The comparatively lower culture positivity of biometric devices in the present study may be because a reasonably higher number, 58 (55.3%), of the fingerprint devices were installed in non-clinical settings of the healthcare facility having the least chances of acquiring and spreading infection via biometric device usage.

Moreover, better health and safety awareness and practices could be another reason for the same although it needs to be evaluated by further KAP study.

Laboratory culture reports revealed that among the positive growth samples, the highest proportion was CONS followed by *Escherichia coli*, *Enterococcus faecalis*, and the least being *Acinetobacter*, MRSA, and *Enterobacter* spp. Similar findings were reported by S. Nirupa et al. (2016), while S. Nancy et al. (2016) reported that one-third of the samples demonstrated the growth of *Staphylococcus aureus*, among which, MRSA were predominant as compared to methicillin-sensitive *Staphylococcus aureus* (MSSA) [7,8]. In another study by Elaine L et al. (2003), regarding the presence of bacterial micro-organisms after handwashing, it was found that the *Staphylococcus aureus* isolation rate was 18.5%, i.e., 40, while that of Gram-negative bacteria was 75.1%, i.e., 168 [9]. Researchers have also reported culture positivity as high as 84 (78.5%) on swab samples collected from other inanimate objects used in hospitals, like stethoscopes, and among them, the highest growth observed was for *Staphylococcus aureus,* 45 (53%), followed by *Pseudomonas aeruginosa,* 16 (19%), *Enterococcus faecalis,* 12 (14.3%), and *Escherichia coli,* 11 (13%) [10]. Samples collected from keyboards and electronic equipment used in the intensive care unit of a hospital in Iran demonstrated growth of CONS on as high as 54 (72%) of cultures by Nazeri et al. and similar isolates of CONS were reported from 25 (92.5%) of the samples collected from computer keyboards by Isaac et al. [11,12]. The evidence produced from the present study and by other researchers in various study settings regarding the presence and pattern of bacterial flora on the surface of biometric print devices reveal a great range and diversity of microorganisms that are transmitted among the users. These may cause a wide range of morbid pathological conditions among the employees responsible for their health concerns. 

Christin R. Blomeke (2007), in their study, reported the rate of survival log of *Staphylococcus aureus* was 0.10 after 20 minutes on the biometric devices and explained the survivability and transferability of these organisms from biometric fingerprinting devices and reported that most of the organisms were transferred in the first 10 minutes after they were located on the surfaces of biometric devices [13].

Considering the above facts in mind, the observation of the present study is that the application of isopropyl alcohol (w/v 70%) on the surface of BFDs for a contact period of 24 hours resulted in a marked reduction of bacterial isolates. These interventional observations prove the bactericidal effect of isopropyl alcohol (w/v 70%) and help in concluding that daily disinfection of biometric fingerprinting devices at workplaces may help the administration to put a barrier to the spread of various micro-organisms among employees thereby providing a better and healthier workplace.

All the supportive studies have shown the growth and transferability of the bacterial flora present on biometric devices or other inanimate devices used in hospitals.

The study's primary strength lies in its robust methodological approach, including a significant sample size and thorough bacteriological analysis, which lend credibility to the findings. The clear demonstration of the efficacy of 70% isopropyl alcohol in reducing bacterial contamination provides valuable insights for infection control protocols.

However, the study has several limitations. The focus on a single healthcare institution may limit the generalizability of the results to other settings with different environmental and operational conditions. Additionally, potential biases in the selection and handling of samples could influence the outcomes. The study period was also relatively short, which may not capture long-term variations in bacterial contamination.

A more comprehensive understanding of this growth pattern could have been achieved through multiple daily swab collections over 5-7 consecutive days. Unfortunately, this aspect was compromised due to insufficient funding for the study.

Future research should address these limitations by including multiple institutions, extending the study duration, and implementing more rigorous sample handling procedures.

## Conclusions

The present study reveals that a definite and considerable presence of bacterial flora on the biometric fingerprinting devices is more prone to carry and transmit microorganisms indirectly from person to person at the workplace among employees. A considerable amount of reduction in bacterial growth on biometric fingerprinting devices after the application of isopropyl alcohol (w/v 70%) helps us to conclude that the surface of biometric fingerprinting devices should be disinfected daily. If not possible, it should be done on an average of every third day to control and minimize the transmission of microorganisms.
